# The potential of circHIPK3 as a biomarker in chronic myeloid leukemia

**DOI:** 10.3389/fonc.2024.1330592

**Published:** 2024-03-05

**Authors:** Eduardo Wandame Gomez, Laura Berti De Paula, Rafael Diogo Weimer, Alessandra Helena da Silva Hellwig, Grazielle Motta Rodrigues, Ana Paula Alegretti, Jarbas Rodrigues de Oliveira

**Affiliations:** ^1^ Laboratory of Cell Biophysics and Inflammation, School of Health and Life Sciences, Pontifícia Universidade Católica do Rio Grande do Sul, Porto Alegre, Brazil; ^2^ Laboratory of Molecular Biology, Laboratory Diagnostic Service, Hospital de Clínicas de Porto Alegre, Porto Alegre, Brazil

**Keywords:** chronic myeloid leukemia, biomarker, ncRNA, circRNA, CircHIPK3

## Abstract

Chronic myeloid leukemia (CML) is a myeloproliferative disorder characterized by leukocytosis and left shift. The primary molecular alteration is the BCR::ABL1, chimeric oncoprotein with tyrosine kinase activity, responsible for the initial oncogenesis of the disease. Therapy of CML was revolutionized with the advent of tyrosine kinase inhibitors, but it is still not considered curative and may present resistance and serious adverse effects. Discoveries in CML inaugurated a new era in cancer treatment and despite all the advances, a new biomarker is needed to detect resistance and adverse effects. Circular RNAs (circRNAs) are a special type of non-coding RNA formed through a process called backsplicing. The majority of circRNAs are derived from protein-coding genes. CircHIPK3 is formed from the second exon of the HIPK3 gene and has been found in various pathologies, including different types of cancer. New approaches have demonstrated the potential of circular RNAs in cancer research, and circHIPK3 has shown promising results. It is often associated with cellular regulatory pathways, suggesting an important role in the molecular dynamics of tumors. The identification of biomarkers is an important tool for therapeutic improvement; thus we review the role of circHIPK3 and its potential as a biomarker in CML.

## 1 Introduction

Considered a model disease for the study of the pathophysiology of cancer due to the progress achieved from the understanding of the molecular mechanisms involved in the initial oncogenesis, Chronic Myeloid Leukemia (CML) was a pioneer in the development of “targeted” therapy and inaugurated a new era in oncological treatment (1). The oncoprotein selective inhibitor has substantially changed the CML outcome, however, some cases develop resistance (2). On the other hand, adverse side effects and toxicities can impact on patient’s quality of life. Despite all the achievements, it is still necessary to improve the care for patients with CML (3). The identification of new biomarkers is a recognized strategy for early detection of diseases, resource optimization, and therapeutic enhancement, capable of predicting severe adverse effects and treatment resistance (4). New approaches in the study of non-coding RNAs (ncRNAs) have shown promising results in various pathologies, especially in cancer (5).

Circular RNAs (circRNAs), are a special type of ncRNAs that are currently receiving special attention and due to their physical characteristic of a covalently closed structure, have a prolonged half-life, and are associated with a variety of mechanisms of action (6). Several studies have investigated the functions of different circRNAs in tumors and circHIPK3 showed consistent results as a biological marker (7). Therefore, our aim in this work is to review the role of circHIPK3 and its potential as a biomarker in CML.

## 2 Chronic myeloid leukemia

Chronic Myeloid Leukemia (CML) is one of the most frequent hematological malignancies, accounting for approximately 15% of leukemia cases (8). It is characterized by a clonal myeloproliferative disorder with leukocytosis, left shift, and splenomegaly due to mutations that alter the hematopoietic stem cell (9). The main molecular alteration in the leukemic stem cell is the presence of the Philadelphia chromosome (Ph), formed through a reciprocal and balanced translocation between the long arms of chromosomes 9 and 22, t(9;22) (q34;q11), which plays a central role in the pathogenesis of CML (8). From this fusion, an oncoprotein with increased tyrosine kinase activity, called BCR::ABL1, is expressed. The increase of activity in this tyrosine kinase triggers the release of cell proliferation effectors and apoptosis inhibitors, through a complex cell signaling network, and its action is considered responsible for the initial oncogenesis of CML (1, 10, 11).

Usually, the disease is discovered during the chronic phase, when clinical signs such as fatigue, weight loss, and fever. A leukocyte count reveals a significant alteration, often with a tenfold increase in relation to normal values. This Leukocytosis is characterized by a massive and escalated presence of the myeloid lineage, but with a discrete presence of blast cells in the peripheral blood (12). The progression of CML is observed through an increase in blast cells in the peripheral blood, suggesting an increase in proliferative activity and a decrease in the ability of cell differentiation. After the chronic phase, there is the accelerated phase, which is characterized by the presence of 10% to 19% blast cells in the peripheral blood. Subsequently, the blast phase occurs, in which the presence of blast cells in the peripheral blood is equal to or greater than 20%. In this phase, disease control is hampered by the accumulation of mutations that often promote resistance to chemotherapy (13).

In the past, the treatment for CML consisted of arsenic derivatives, busulfan, and hydroxyurea. Afterward, it progressed to the use of interferon-alpha and bone marrow transplantation (9, 12, 14, 15). However, with the development of targeted therapy, the treatment for CML was revolutionized with the advent of the first tyrosine kinase inhibitor (TKI), imatinib mesylate, formerly known as STI571. The average 5-year survival rate after diagnosis increased from 22% to 70%, and many patients undergoing TKI treatment, now, have a relatively normal life expectancy. At the same time, new generations of TKIs have been developed with the aim of limiting adverse effects and tumor resistance. Despite significant advancements, TKI therapy is still not considered curative, and in some cases, severe adverse effects make treatment impractical. Naturally resistant tumor cells or those that acquire resistance over time are also observed (16–18). Therefore, it is recommended to regularly monitor BCR-ABL1 transcript levels in patients treated with TKIs to evaluate the molecular response to treatment (18). Currently, there is no biomarker capable of early detection of resistance and adverse effects (19).

## 3 Circular RNAs

Circular RNAs are a special type of non-coding RNA formed by a covalently closed ring structure. Their circular conformation was first described over 40 years ago in different viruses and later found in eukaryotes, however, their biological functions remained unknown for a long period of time (20). Most CircRNAs are expressed from known protein-coding genes, formed through a non-canonical splicing event called backsplicing. CircRNAs result from the fusion of splice sites, creating abundant, conserved, and stable covalently closed circles. Due to their conformation, they have a long half-life (~20 hours) and are degraded by endonucleases, especially RNase L (7, 21).

According to the sequence from which they are derived - exon, intron, or both - circRNAs can be divided into ecircRNAs, EIciRNAs, and CiRNAs (2). EcircRNAs can be derived from a single exon or from the fusion of different exons, sometimes containing exons that are not present in linear transcripts and normally are in the cytoplasm of the cell (7). The EIciRNAs are formed by the fusion of exons and introns, resulting from intron retention, and the ciRNAs are formed from introns, due to a failure in the debranching of intronic loops during canonical splicing (22). They possess unique characteristics, such as high stability and tissue specificity, and are abundant in eukaryotic cells, being able to play a regulatory role in transcriptional and post-transcriptional levels (2).

The mechanisms of action of circRNAs include interacting with microRNAs (miRNAs) through binding and inhibition of their activities, transcriptional regulation, interactions with proteins, and their own translation (2, 23). The inhibition of miRNAs, also known as the sponge mechanism, occurs with the retention of miRNAs in the complementary binding sites of circRNAs, preventing their action on the target messenger RNA (mRNA) (24). Transcriptional regulation occurs through the interaction of circRNAs with promoter regions by binding to U1 snRNP (small nuclear ribonucleoprotein U1) and Pol II (RNA polymerase II), which are important factors involved in mRNA processing (25). CirRNAs can also interfere with the interaction between different proteins by bringing their interaction sites closer or further apart, act in protein recruitment, and even have their own sequence translated (7).

The various forms of interaction with the important signaling pathways have evidenced the potential of circRNAs as a biomarker in different types of tumors for early diagnosis, detection of metastases, prognosis, and resistance to treatment. This is considered very promising, and recently several studies have shown important results (2, 23).

## 4 Circular RNA HIPK3

CircHIPK3 is a EcircRNA derived from the gene encoding homeodomain-interacting protein kinase 3 (HIPK3) that is located on chromosome 11p3 in humans. It consists of 7,551 pairs of bases and belongs to a family of protein kinases composed of four serine-threonine nuclear kinases: HIPK1, HIPK2, HIPK3, and HIPK4 (26). These kinases are evolutionarily conserved and share a similar basic structure. HIPKs participate in cellular regulatory mechanisms by phosphorylating various transcriptional regulators. Among them, they play important roles in processes related to carcinogenesis such as chromatin modifiers, proliferation, apoptosis, DNA damage response, oxidative stress, and cellular development (27, 28).

### 4.1 Generation of circHIPK3

**Figure 1:**
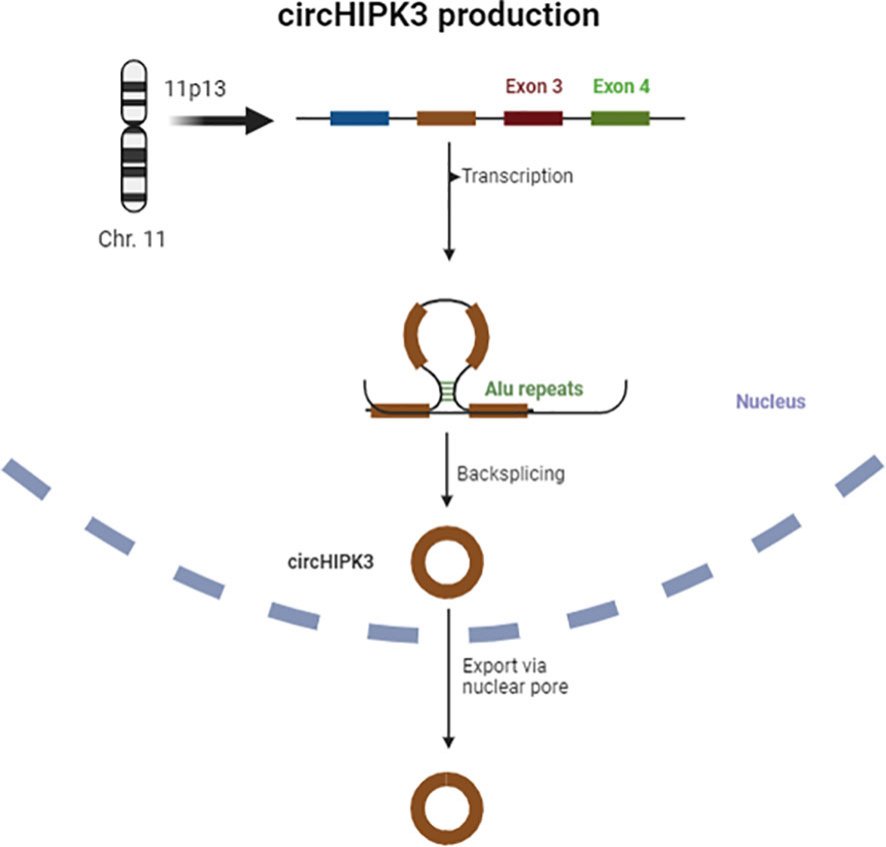
Scheme of circHIPK3 production.

The sequencing of the HIPK3 gene has shown that its second exon, coupled with long flanking introns containing Alu repeats, is complementary at both ends and promotes cyclic characteristics, giving rise to a circRNA, also known as circHIPK3 (29). **Figure 1** provides a schematic representation of circHIPK3 production.

The circHIPK3 derived from exon 2 is the most abundant form, but a study by Qiupeng Zheng et al. has shown that there are different isoforms of this circRNA and variations in its concentration across different tissues (30). Mainly located in the cytoplasm, circHIPK3 is abundantly expressed in various tissues such as cardiac, pulmonary, and intestinal tissues, and has been consistently associated with neurological disorders, cancer, cardiovascular, and inflammatory diseases (20, 27, 31, 32).

### 4.2 Mechanism of action of circHIPK3

The mechanism of action of circHIPK3 in various physiological and pathological processes described so far is based on its ability to intervene in gene regulation through the sponge activity exerted on multiple miRNAs, resulting in their inactivation. It has been experimentally demonstrated by Zeng et al. that circHIPK3 is able to capture 9 miRNAs through 18 potential places of connection. By utilizing bioinformatics tools, such as CircInteractome, it is predicted that circHIPK3 can potentially inactivate 42 miRNAs through its sponge action (27, 33–35). M. Bai et al. demonstrated that the binding capacity of circHIPK3 and consequent inactivation of the target miRNAs is not restricted to the complete complementarity of nucleotide sequences, a partial binding can also result in miRNA retention and blockage of its effects, justifying the experimental confirmation of some target miRNAs not computationally predicted (31). Target miRNAs of circHIPK3 are listed in **Table 1**, both those predicted by bioinformatics tools and those confirmed experimentally (27). Not all confirmed miRNAs were predicted. The miRNAs that were both predicted and confirmed are highlighted.

**Table 1:** Predicted and confirmed circHIPK3 target miRNAs (highlighted).

Prediction miRNAs				Confirmed miRNAs		
miR-149	miR-513a-3p	miR-668		miR-7	miR-326	miR-561
miR-192	miR-515-5p	miR-766		miR-29a	miR-330-5p	miR-582-3p
miR-215	miR-558	miR-1178		miR-29b	miR-338-3p	miR-584
miR-326	miR-561	miR-1179		miR-107	miR-379	miR-599
miR-330-5p	miR-579	miR-1231		miR-124	miR-381-3p	miR-637
miR-338-3p	miR-580	miR-1243		miR-124-3p	miR-421	miR-653-5p
miR-346	miR-584	miR-1250		miR-149	miR-448	miR-654
miR-375	miR-599	miR-1278		miR-152	miR-485-3p	miR-876-5p
miR-377	miR-606	miR-1283		miR-192	miR-506	miR-1207-5p
miR-382	miR-607	miR-1286		miR-193a	miR-508-3p	miR-1286
miR-485-3p	miR-619	miR-1290		miR-212	miR-524-5p	miR-4288
miR-490-5p	miR-637	miR-1294		miR-215-3p	mir-558	miR-4524-5p
miR-495	miR-640	miR-1305				
miR-508-3p	miR-653	miR-1825				

This mechanism of action that involves the inactivation or degradation of miRNAs is important in the course of various pathologies. In myocardial ischemia, Bai et al. demonstrated that circHIPK3 inhibits proliferative capacity and induces heart cells to apoptosis by binding with miR-124-3p (31). Chaofang Lian et al. demonstrated that circHIPK3, by binding with miR-561 and miR-192, activates NLRP3 macrophage inflammation and TLR4 pathway in gouty arthritis (32). Regarding the oncogenic process, several studies have shown that the regulation exerted by circHIPK3 by binding with multiple miRNAs plays an important role in different types of cancer, such as breast (36), pancreas (37), lung (38), gut (39), liver (40), brain (41), esophagus (33), renal (42) and blood (43). Some of these studies investigated the cellular signaling pathways involved in circHIPK3 action and, predominantly, increased levels of circHIPK3 combined with miRNA binding result in tumor progression (27). Qi et al. described the role of circHIPK3 in breast cancer, concluding that its interaction with miR-326 promotes tumor proliferation, migration, and invasion (36). In pancreatic cancer, Liu et al. correlated tumor resistance to chemotherapy by linking circHIPK3 with miR-330-5p (37).

In experiments with lung cancer cells, Chen et al. attributed the influence of circHIPK3 to the miR-124-3p-STAT3-PRKAA/AMPKa axis as an autophagy regulator, an important cellular mechanism for the removal of abnormal and undesirable proteins (44). Hongqian Yin and Xia Cui demonstrated that the silencing of circHIPK3 in glioma cells can promote sensitivity to treatment with Temozolomide, modulating proliferation, metastasis, and apoptosis through interaction with miR-524-5p/KIF2A, mediated via PIK3/AKT (45). Feng Gu et al. identified that the silencing of circHIPK3 inhibits the progression of lung cancer by the sponge mechanism that, by binding with miR-381-3p, inactivates the AKT/mTOR (46) signaling pathway. Da Yao et al. demonstrated that circHIPK3 absorbs miR-124 and promotes AKT3 expression in squamous cells of esophageal carcinoma (33). Enrico Gaffo et al. investigated the differential expression of circular RNAs in pediatric acute lymphoblastic leukemia (ALL) and found an increased, marked, and generalized expression of circHIPK3 in pediatric B-precursor acute lymphoblastic leukemia (43).

### 4.3 The dualistic role of circHIPK3 in cancer

**Figure 2:**
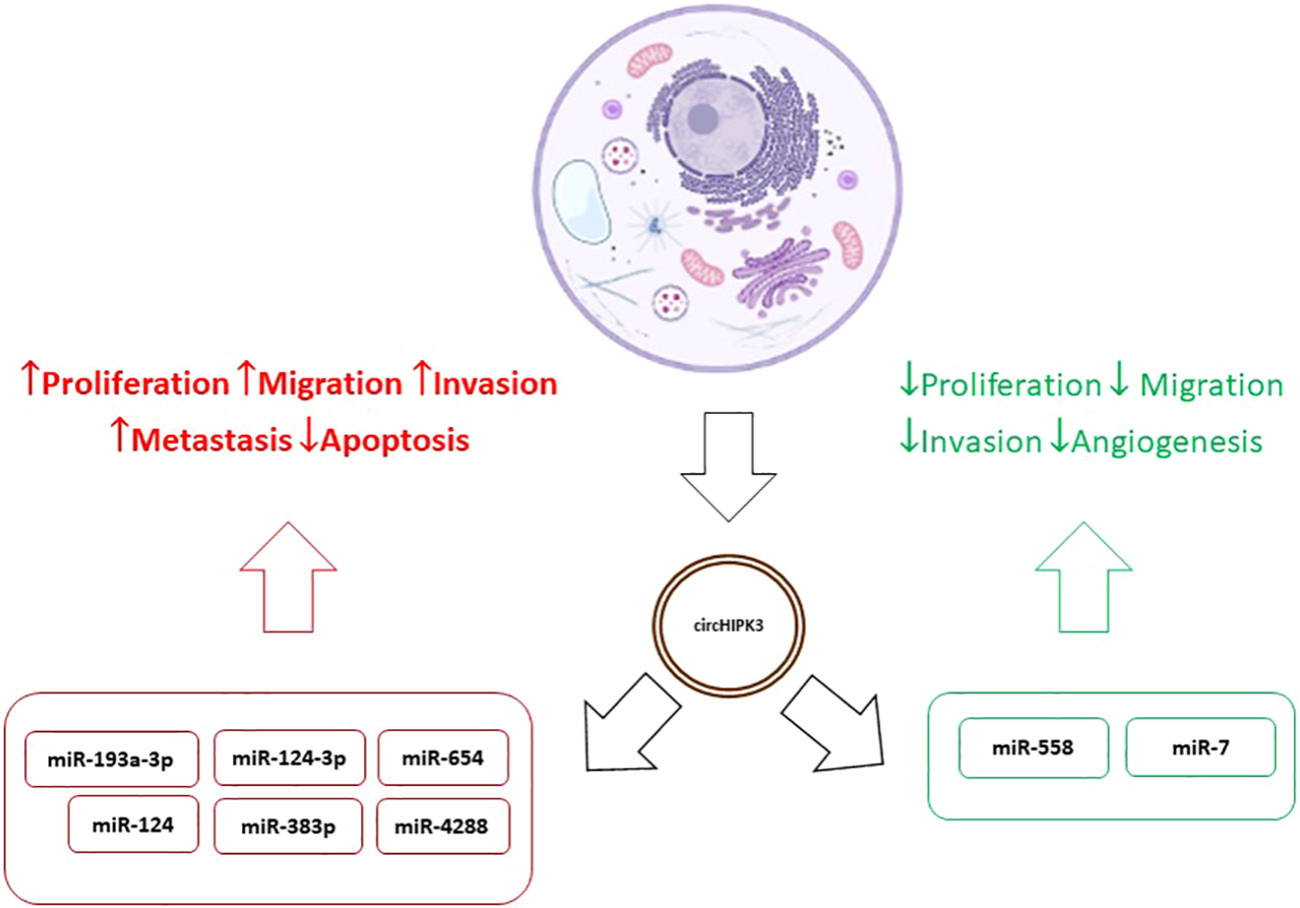
Dualistic mechanisms of circHIPK3 action in cancer.

Results relating increased circHIPK3 values to tumor progression and resistance to treatment were found in several studies (39, 41, 44–46). On the other hand, Yawei Li et al. concluded that overexpression of circHIPK3 inactivates miR-558 by the sponge mechanism, inhibiting migration, invasion, and angiogenesis of bladder cancer cells (47) and Mao Xiao-Long et al. indicated that high levels of circHIPK3 significantly suppress the proliferation, migration, and invasion of osteosarcoma cells (48). Zeyu Wei et al. reviewed the role of circHIPK3 in various cancer types and its overexpression was associated with development, progression, metastasis, and multidrug resistance. In addition, as already mentioned, tumor suppression effects were observed in certain tumors such as the bladder. At the same time, in kidney cancer, osteosarcoma, and ovarian cancer expression patterns and functions of circHIPK3 were contradictory. This behavior suggests, disregarding the experimental limitations of each study, that circHIPK3 may present differentiated levels and effects in different cell lines in the same tumor type (49). This duality, added to the variety of mechanisms of action of circHIPK3, demonstrates the complexity of their interactions in tumor cells and indicates the need for further studies to discover the meaning of these variations, diagnosis, and treatment of the various types of tumors. So, the different levels of expression found in various types of cancer may be related to the type of cancer and level of tumor progression (46–49). **Figure 2** shows the dualistic mechanisms of circHIPK3 action in cancer.

## 5 The role of circHIPK3 in CML

As in several types of cancer, in CML the pathways of cellular regulation are altered, favoring mechanisms of proliferation and apoptotic resistance (49). Thus, the molecular interactions of oncoproteins are in constant research for the development of new therapeutic targets, diagnostic markers, prognoses, and chemotherapy resistance (50). With the advances provided by revolutionary discoveries in CML, such as the Philadelphia chromosome and the first cancer-specific target therapy, the disease is considered a model for understanding the mechanisms involved in cancer pathogenesis (51).

Therefore, many studies have advanced in understanding this disease and demonstrated that the molecular signaling of the oncoprotein BCR::ABL is quite complex (52), and its precursors, the genes Abl 1 and BCR, are formed by 11 and 23 exons and can merge in several ways, causing multidomain chimeric oncoproteins with differentiated molecular weights and associated with different leukemic tumors. In CML, the main oncoprotein, in approximately 98% of cases, is the major BCR (M-BCR) also called BCR::ABL1 P210 (52). Its role is not yet fully understood, however, in this fusion oncoprotein, there is a potent intensification of tyrosine kinase activity observed in the normal gene Abl1. It promotes dimerization or tetramerization, resulting in autophosphorylation of other Abl1 sites that lead to more binding sites for the homologous Src 2 (SH2) anchoring domain in several proteins, activating a multitude of signaling pathways. Thus, BCR::ABL1 oncoprotein can recruit and activate several signal transducers downstream through SH2 in these proteins. Similarly, an inhibitory change in the negative regulatory domain SH3 is described through the first sequences of exon BCR (53). Together, the regulatory change in SH2 and SH3 promotes inhibition of apoptosis, cell transformation, and self-renewal capacity (54). Parallel to the complex molecular interaction triggered by the presence of the oncoprotein BCR::ABL1, it is described, in several pathologies, the action of circHIPK3 predominantly through the inactivation of miRNAs by the sponge mechanism (27) and in pathways common to several types of cancer, including the CLM. These findings and the growing discovery of interactions of circHIPK3 with different pathways through the circHIPK3/miRNA/mRNA pathway indicate a possible role of circHIPK3 in pathways of cell regulation compromised with CML pathogenesis.

Several target miRNAs of circHIPK3 have been shown to interfere with the gene expression of a variety of proteins involved in various signaling pathways. Sometimes, the same signaling route is deregulated in different ways, such as PI3K/AKT, which is augmented by the interaction circHIPK3/miR-637/NUPR1, HIPK3/miR-7/IGF1R, circHIPK3/miR-124/AKT3 and circHK3/miR-193a/HMGB1 (33, 35, 55, 56). Liu and collaborators described the influence of circHIPK3 on the Wnt/ß-catenin pathway by unknown mechanisms and Zeng et al. attributed it to the circHIPK3/miR-7/YY1 interaction down-stream deregulation of the same pathway (35, 57). The Hippo/YAP pathway was also deregulated from the circHIPK3/miR-381-3p/YAP1 interaction (58). Chen et al. described the deregulation of the PRKAA/AMPKα pathway from the circHIPK3/miR-124-3p/STAT3 interaction (44). Deregulation of the AKT/mTOR pathway was attributed to the interaction circHIPK3/miR-381-3p with an unknown target gene (46). Zeng described the influence of circHIPK3/miR-7/EGFR on the deregulation of the MEK/ERK pathway (35) and the circHIPK3/miR-637/STAT3 mechanism deregulated the Bcl2/Beclin1 pathway (59).

Researching in patients with CML, Feng et al. found an increased expression of circHIPK3 in mononuclear cells and serum of CML patients compared to healthy donors. The correlation between the highest levels of circHIPK3 and the worst prognosis was also observed. The experiments indicated that the mechanism of sponge action of circHIPK3 on miR-124, already observed in other studies, influenced the targets of miR-124 B4GALT1, and nuclear factor-kB (NFkB) p65, but not in IGF2BP3 (34). Hong Che and colleagues investigated to understand one of the main problems related to CML, the resistance to treatment with imatinib. Their findings revealed that the miR-326/PPFIA1 axis plays a significant role in contributing to this resistance through circ_0080145 modulation (60). This discovery underscores the potential of circHIPK3 as a marker for identifying chemotherapy resistance in CML since circHIPK3/miR-326 binding was evidenced by Qi et al. in breast cancer and linked to cell proliferation, migration, and invasion (36).

## 6 Conclusion

Determination of the properties and functions of circRNAs and their importance in the pathophysiology of cancer is a recent topic that has been driven by new sequencing technologies combined with an increasing interest in the study of pathways circRNAs/miRNAs/mRNAS/proteins. Evidence regarding the association between circHIPK3 levels and various pathologies, particularly different types of tumors, suggests that these molecules play an important role in the molecular dynamics of tumors. The expression levels of circHIPK3 in different types of cancer are predominantly found to be overexpressed while suppressing certain microRNAs, and its pro-oncogenic consequences suggest that imbalances in its activity can be detrimental to cellular homeostasis.

The role of circHIPK3 in leukemia, particularly in CML, which is considered a model disease for cancer studies, needs further exploration. It is essential to clarify all the effects and interactions of circHIPK3 in molecular signaling pathways, especially those related to proteins with tyrosine kinase activity, to determine its importance as a biomarker. Advances in studies, fundamentally in the dynamics of the tumor microenvironment, which is considered a key element for the extinction of leukemic stem cells, may determine the relevance of circHIPK3 in CML.

## Author contributions

EG: Conceptualization, Writing – original draft, Writing – review & editing. LP: Writing – original draft, Writing – review & editing. RW: Writing – original draft, Writing – review & editing. AH: Writing – original draft, Writing – review & editing. GR: Writing – original draft, Writing – review & editing. AA: Writing – original draft, Writing – review & editing. JO: Conceptualization, Supervision, Writing – original draft, Writing – review & editing.
